# Role of Kisspeptin on Hypothalamic-Pituitary-Gonadal Pathology and Its Effect on Reproduction

**DOI:** 10.7759/cureus.17600

**Published:** 2021-08-31

**Authors:** Jaskamal Padda, Khizer Khalid, Amir Moosa, Mohammad Syam, Varsha Kakani, Urooj Imdad, Dina Ismail, Ayden Charlene Cooper, Gutteridge Jean-Charles

**Affiliations:** 1 Internal Medicine, JC Medical Center, Orlando, USA; 2 Internal Medicine, Advent Health & Orlando Health Hospital, Orlando, USA

**Keywords:** kisspeptin, hypothalamic-pituitary-gonadal axis, reproduction, puberty, infertiliy, gpr54, kiss1r, kiss1 gene

## Abstract

Kisspeptin is a neuropeptide that plays a significant role in human reproduction by its action on the hypothalamic-pituitary-gonadal (HPG) axis and functions through a G-protein-coupled receptor called G-protein-coupled receptor 54/kisspeptin 1 receptor (GPR54/KISS1R). It is encoded by the kisspeptin 1 (KISS1) gene that is mainly expressed in the hypothalamus. Kisspeptins are also recognized as vital aspects of maturation and proper function of the reproductive system in both males and females. It also plays its role in the onset of puberty, sexual patterns, desires, ovum development in women, sperm quality in men, feedback mechanisms, pregnancy, and lactation. Studies proved the pathological role of kisspeptin dysregulation in disorders like polycystic ovarian syndrome (PCOS) and infertility. Mutations in the KISS1 gene also contribute to precocious puberty or hypogonadotropic hypogonadism, depending upon the nature of mutations. Levels of kisspeptin also aid in the identification of a few pregnancy-related complications like preeclampsia, intrauterine growth restriction, and act as a marker of miscarriage. Due to the wide range of effects that kisspeptin has on the reproductive axis, investigations are being carried out to develop it as a diagnostic marker, treat diseases like hypogonadism and PCOS, and solve infertility issues.

## Introduction and background

**Figure 1:**
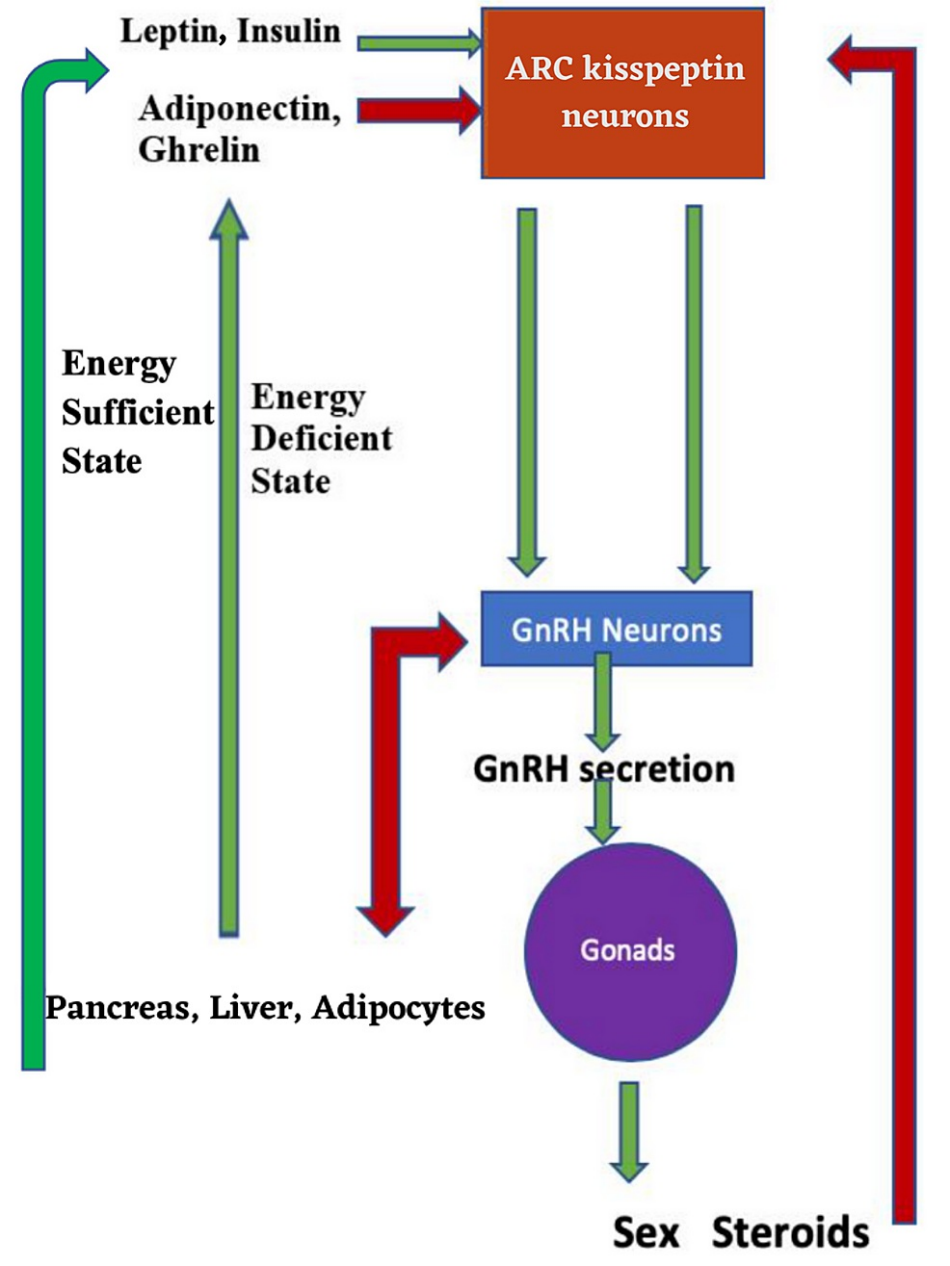
Role of kisspeptin in the regulation of the reproductive cycle of humans. ARC - Arcuate nucleus, GnRH - Gonadotropin-releasing hormone Estrogen, progesterone, and testosterone, which are sex hormones, have a negative feedback control on kisspeptin neurons. Kisspeptin neurons are also influenced by leptin and insulin (excitatory) as well as ghrelin and adiponectin (inhibitory). The excitatory inputs are represented by green arrows and inhibitory inputs by red arrows. Copyright/License: This figure is from an open-access article distributed under the terms and conditions of the Creative Commons Attribution license. (http://creativecommons.org/licenses/by/4.0). Source: Kant R, Meena MK: Role of kisspeptin in puberty in humans [2].

Kisspeptin is a peptide hormone by structure that has a vital role in the reproductive cycle of humans through its influence on the hypothalamic-pituitary-gonadal (HPG) axis. The arcuate nucleus and periventricular nucleus of the third ventricle, located in the hypothalamus, are involved in the production of this neuropeptide. Kisspeptin, through its receptor KISS1r (kisspeptin 1 receptor), stimulates the production of gonadotropin-releasing hormone (GnRH). Positive and negative feedback mechanisms regulate this intricate balance through sex steroids [1]. Figure 1 elaborates on this intricate feedback mechanism [2].

Kisspeptin and its receptor system are also found in other organs, such as the ovary of mature female humans where it plays a critical role in developing a mature ovum and contributes to fertility. This effect is further supported by the fact that mutations involved in the kisspeptin pathway may lead to infertility problems [3]. Apart from the physiological role of kisspeptin and its receptor in the body, it is also involved in pathological conditions such as polycystic ovary syndrome (PCOS) and infertility issues associated with hyperprolactinemia due to prolactinoma or other similar conditions [4]. There are future prospects to utilize kisspeptin analogs and antagonists for issues like hypogonadism and precocious puberty, and studies are being conducted to evaluate kisspeptin usage as a disease marker [5]. Researchers also have a great interest in the role of kisspeptin as an antineoplastic hormone secreted by organs other than the brain, i.e., the placenta [6]. Additionally, kisspeptin and related neuropeptides are being investigated for their possible role in influencing sexual patterns and desires, and hence contributing to libido [1]. Kisspeptin and its receptor have a role of immense importance in the reproductive system uncovered recently, and researchers are utilizing this opportunity for the betterment of public health.

## Review

Kisspeptin and its receptor

Kisspeptin is a family of neuropeptide products of the KISS1 gene (gene ID:3814), located at 1q32 [7, 8]. The KISS1 gene encodes 138 amino acid pre-prohormones, the first 19 amino acids contribute to signal peptides. The pre-prohormone is sent to the endoplasmic reticulum and then cleaved into a series of peptides of 13, 14, and 54 amino acids in length [9]. These neuropeptides have the same c-terminus and are biologically active. Kisspeptin was initially termed metastin, as it was first discovered as a metastasis inhibitor in melanoma cells [10]. Kisspeptin neurons are found in the infundibular nucleus and preoptic area in the human brain [11].

The kisspeptin receptor is a G-protein-coupled receptor discovered in 1999. It was initially known as G-protein-coupled receptor 54 (GPR54), but after discovering its strong affinity to kisspeptin, it became known as the KISS1R [12]. Mutations in the KISS1R gene (gene ID: 9291), located at 19p13.3, were associated with hypogonadotropic hypogonadism in humans, which suggests that kisspeptin and its receptor play an important part in the regulation of the HPG axis [13, 14]. The KISS1R is expressed in many brain (pons, midbrain, thalamus, hypothalamus, hippocampus, amygdala, cortex, frontal cortex, and striatum) and peripheral (liver, pancreas, and intestine) regions. The areas with particularly high expression are the placenta, pituitary gland, pancreas, gonadotrophs, testicles, ovaries, and spinal cord, further cementing the endocrine and reproductive function of the KISS1/KISS1R system [9, 15-17].

Control of kisspeptin in reproduction

Kisspeptin is a neuromodulator that acts as an important regulator of gonadotropin secretion. It plays a part in both men and women [18]. Kisspeptin is expressed with other neuropeptides like neurokinin B and dynorphin; together, they regulate hypothalamic reproduction control. These are together called the Kisspeptin-Neurokinin B-Dynorphin (KNDy) neurons [19]. Kisspeptin plays a vital role in the onset of puberty, maintaining the normal reproductive function, coordinating sex steroid feedback on the reproductive axis, and gender differentiation in the brain. The onset of puberty is dependent on various genetic and environmental factors. Studies show that impaired pubertal development occurs if there is no proper interaction between kisspeptins and their receptors. This is explained by the studies showing that deletions or mutations in the KISS1 receptor genes are associated with abnormal pubertal development. Moreover, activating mutations in the KISS1 receptor genes are associated with precocious puberty [18, 20].

In females, hypothalamic kisspeptin acts on GnRH neurons and mediates the release of the gonadotropins luteinizing hormone (LH) and follicle-stimulating hormone (FSH) which stimulate sex hormone synthesis. It is shown that kisspeptin exerts both direct and indirect actions on the HPG axis. The direct effect is on the pituitary gonadotrophs to release LH and FSH. The indirect effect is the primary physiological pathway. It involves the action on the hypothalamic GnRH system, leading to GnRH secretion and thereby LH and FSH. There are variations in GnRH pulsatility in the menstrual cycle, which changes at various times of the cycle. The arcuate nucleus is the site for GnRH pulse generation, and kisspeptin acts here to modulate GnRH secretion. Increased estrogen at the end of the follicular phase of the menstrual cycle activates KISS1 neurons, which increases the GnRH pulse frequency (greater than one pulse per hour) and amplitude. This increase causes LH surge and ovulation. So, kisspeptin is partly responsible for ovulation via causing LH surge. On the other hand, the slow frequency GnRH pulse (less than one pulse per two-three hours) favors FSH secretion [18,20].

In addition to its effect on the hypothalamus and pituitary gland, kisspeptin also acts on the ovaries. The LH surge at the end of the follicular phase of the menstrual cycle causes an increase in brain-derived neurotrophic factor (BDNF) in the granulosa cells of the ovary. This BDNF signaling to the oocyte plays a crucial part in oocyte developmental compromise. The role of BDNF is proved through various studies conducted on the mouse, porcine, bovine, and human ovaries [21].

Kisspeptin plays a crucial part in estrogen-mediated negative and positive feedback on LH secretion. There is negative feedback on LH secretion during the start of the follicular phase of the menstrual cycle. It later changes to positive feedback by the end of the follicular phase. Negative feedback of estrogen is mediated by kisspeptin in the infundibular nucleus. However, estrogen mediates its negative feedback by suppressing the kisspeptin and neurokinin B release from the KNDy neurons. This, in turn, reduces the stimulatory input to the GnRH neurons. Estrogen switches from negative to positive feedback in the late follicular phase to induce LH surge and triggers ovulation. This positive feedback mechanism is still unclear, although some studies have shown that the action of KNDy neurons is similar to negative feedback [18]. Both positive and negative feedback mechanisms occur through the estrogen receptor α (ER α) signaling pathway. It is known that GnRH neurons lack ER α. This shows that some intermediate pathway serves as the missing link and conveys the effects of estrogen onto the GnRH neurons. Further research showed that the missing link in this pathway is the KISS1 neurons. The KISS1 neurons organize during the early periods of sex differentiation. Later, during puberty also, they undergo complex neuroanatomical modification [22].

There is sexual dimorphism in the kisspeptin pathway in humans. There is variation in the distribution, expression, and number of kisspeptin fibers and cell bodies in males and females. In females, the hypothalamus has more fibers in the infundibular nucleus and periventricular zone than males [23].

In males, kisspeptin has both central and peripheral actions similar to that in females. As part of the central action, it acts on the GnRH neurons in the hypothalamus, which stimulates GnRH secretion, thereby LH and FSH. Males with the deletion or mutation in the KISS1 receptor gene are shown to develop hypogonadotropic hypogonadism. As a part of the peripheral action, it acts on the testes. Studies have shown the distribution and activity of KISS1 in the human testes. The central action alone is insufficient, and the complementary effects on both the testes and hypothalamus aid in proper reproductive function. Research shows that there is a direct effect of kisspeptin on the male gamete. This is established by the presence of the KISS1 receptor in the spermatozoa, mainly in the head (post-acrosomal region), neck, and flagellum in humans [24]. KISS1 is also distributed in seminal plasma [24]. Kisspeptin effect on sperm function is translated by increasing intracellular Ca2+ in spermatozoa in addition to increasing sperm motility with a transient hyperactivation of sperm [24]. A recent study by Zou et al. conducted among 666 Chinese student volunteers showed a positive association between total seminal plasma kisspeptin and semen quality, which was determined by the concentration of the sperm, total sperm count, and motile sperm count (P<0.01) [25]

Pathological effect of kisspeptin on HPG

Kisspeptin regulates various pathways, including follicular development, oocyte formation, ovulation, ovarian steroidogenesis, embryo implantation, and placenta in females. In addition, it plays an essential part in spermatogenesis, spermatozoa function, motility, and testicular steroidogenesis in males [26].

GPR54/KISS1R system has a leading role in triggering elevated gonadotropin secretion levels during puberty and initiating reproductive function. Therefore, mild to severe restricted sexual transformation to hypogonadism can occur due to mutations in GPR54/KISS1R [5]. Biallelic complete loss-of-function mutations of the KISS1R gene is known as the most unfavorable phenotype generating from a homozygous 155 base pair deletion, homozygous frameshift deletion, homozygous splice acceptor site mutation, homozygous and F272S missense mutation, and compound heterozygous mutation R331X/X399R and R297L (mild impairment)/C223R (impair significantly) [27]. Interestingly, the entire biallelic mutation does not manifest complete GnRH deficiency, some endogenous GnRH is detected in those mutations. Furthermore, inconsistency within siblings was discovered in KISS1R mutation, though the exact reason is still unknown [27].

Additionally, the involvement of heterozygous mutations in one or multiple genes has been notifiable for GnRH deficiency, such as prokinectin receptor 2 (PROKR2), the most common monoallelic mutation seen in the human body [27]. Patients with central precocious puberty revealed a gain-of-function mutation in the C terminal tail of KISS1R (R386P). Recent analysis indicates that R386P extends kisspeptin sensitivity by lessening the breakdown of its receptor. In addition, two KISS1R mutations (P74S and H90D) were recently identified with idiopathic central precocious puberty [27].

GnRH is mandatory for testicular descent and penile growth as part of fetal development. As a result, alteration in GPR54 causes small penis as well as cryptorchidism. Kisspeptins provoke GnRH secretion in puberty, and their loss-of-function mutations lead to a lack of pubertal growth [18]. Therefore, infertility occurs in both genders due to altered GPR54/KISS1R. Hence, hormone replacement therapy (specifically GnRH) restores reproductive function. Kisspeptin and GPR54 are located in the sperm head and around the neck, respectively, which the western blot detects. A variable amount of kisspeptin is evident in different stages of fertility. Besides, male patients retaining KISS1R mutation conform to external hormonal therapy and can gain reproductivity [28].

Kisspeptin exerts a pivotal stimulatory role in LH preovulatory surge resulting in defoliation of ovum. Anatomically kisspeptin neurons are linked with GnRH neurons representing GPR54, and the trial revealed a substantial stimulatory effect of kisspeptin in gonadotrophin secretion by either central or peripheral administration. In humans as well as in animals (sheep and monkeys), a low dose of kisspeptin in the intracerebroventricular space markedly increased LH and FSH secretion. Furthermore, PCOS is intimately connected to the elevated level of KISS1R expression and demonstrates positive feedback of kisspeptin and elevated LH levels, which is prominent in the pathophysiology of PCOS [29].

Kisspeptin is known as metastin because it helps prevent metastasis in melanoma and breast cancer owing to its ability to inhibit cell invasion, altering cellular motility and adhesion. These criteria made kisspeptin crucial in embryo implantation, later in in-vitro fertilization (IVF)/assisted reproduction technology. The nidation depends on the degree of trophoblast infiltration into the uterine extracellular matrix where kisspeptin combined with other proinflammatory cytokines, notably tumor necrosis factor-alpha, blocks invasion by stimulating apoptosis [5]. As a result, KISS1 plays a part in controlling the rate of syncytiotrophoblast cell invasion and angiogenesis, ensuring that early placentation occurs in a controlled and sequential manner [30]. Moreover, KISS1R and GPR54 are predominantly expressed in the trophoblast of the first-trimester placenta, where the invasive capacity is at its highest. On the contrary, KISS1R and GPR54 are poorly expressed in the full-term gestation placenta [30]. We can deduce that KISS1 has a similar part in the inhibition of cell migration in both early pregnancy and tumor metastasis [30].

Up to a 200-fold increase of kisspeptin is evident in the third-trimester pregnancy compared to nonpregnant women. Plasma kisspeptin below 1630 pmol/L in the first trimester is considered a miscarriage marker since those who suffer from miscarriages have a kisspeptin level that is 60% lower than those with uneventful gestations. Furthermore, reduced kisspeptin levels in the second trimester (16-20 weeks) were linked to intrauterine growth restriction, and lower levels at the beginning or end of pregnancy were related to preeclampsia. In the end, the combination of intrauterine condition, preterm, epigenetic mechanisms, and parental planning should be considered seriously because of disease development in the fetus and in later life [5].

Treatment

Kisspeptin is being investigated as a biological biomarker in women undergoing IVF, those who have cancer, and to probe fertility status. This is why it is important to better understand kisspeptin signaling in the female reproductive life cycle, especially in patients with PCOS and hypertensive disease in pregnancy. Kisspeptin metabolic effect on obesity and insulin release also needs further investigation [5]. It is still not clear which indications one would consider administering exogenous kisspeptin as kisspeptin dosage and forms are still debatable. However, despite its importance, any diagnostic or therapeutic use of kisspeptin has largely been investigational and under review and warrants more experimental studies and clinical trials to explore the promising possibilities.

## Conclusions

Kisspeptin, a neuromodulator, is produced mainly in the arcuate and periventricular nuclei of the third ventricle in the hypothalamus. It acts through the KISS1 receptor and stimulates the production of GnRH. Kisspeptin plays a crucial role in the onset of puberty, feedback mechanisms, development of the ovum, quality of semen, sexual desires, pregnancy, and lactation. Kisspeptin is involved in steroidogenesis in both the ovaries and testes. Kisspeptin has effects along the entire reproductive axis starting from the hypothalamus to the ovary, as it aids in ovulation and plays an important role in oocyte development. It is also responsible for the positive and negative feedback mechanisms of estrogen on LH. Its effect on the testes is crucial for testicular descent and penile growth in the developmental stages and it enhances the quality of semen during the reproductive years. Apart from the reproductive function, it is also known as metastin as it is known to prevent the metastasis of melanoma and breast cancer. Mutations in the KISS1R gene have shown unfavorable reproductive outcomes in humans. Research has been started to effectively use the exogenous kisspeptin analogs and antagonists in the treatment of disorders. Further studies are needed to understand the dosage and route of administration. Because of its effect on embryo implantation, it is also studied to have a useful role in IVF. Future research is mandatory in exploring the diagnostic and therapeutic application of kisspeptin that will lead to better reproductive health in humans.
